# Modulation of the Nrf2/HO‐1 Pathway‐ and Apoptosis‐Related Genes Following 5‐hydroxymethylfurfural Induced Mouse Liver Injury

**DOI:** 10.1002/jbt.70385

**Published:** 2025-06-30

**Authors:** Hatice Kurtel, Yasemin Aydin, Banu Orta Yilmaz

**Affiliations:** ^1^ Institute of Graduate Studies in Science and Engineering, Department of Biology Istanbul University Istanbul Turkey; ^2^ Department of Biology, Faculty of Science Istanbul University Istanbul Turkey

**Keywords:** 5‐hydroxymethylfurfural, apoptosis, hepatotoxicity, mice liver, oxidative stress

## Abstract

Food contaminants released from heat‐treated foods have been an issue of global investigation in recent years. The risk and toxicity assessment of 5‐hydroxymethylfurfural (HMF), which is mostly exposed to through food consumption, is also of great importance. Studies have revealed the toxicity of HMF on various tissues and systems. However, there are not enough studies on the toxic effects of HMF on the liver. This study applied different doses of HMF (30 and 300 mg/kg) to adult mice for 21 days. Liver tissues obtained from mice exposed to HMF were examined histologically and histopathologically. The investigation of oxidative damage in HMF‐induced liver tissue involved the spectrophotometric measurement of malondialdehyde, hydroxyl radicals, and hydrogen peroxide levels and the activities of superoxide dismutase, catalase, glutathione peroxidase, glutathione, gamma glutamyl transpeptidase, and glutathione‐S‐transferase. The expression levels of genes associated with the Nrf2/Keap1/HO‐1 signaling pathway were examined. Furthermore, oxidative stress‐related genes and important genes in the apoptotic pathway were analyzed for their expression levels. The findings indicated that HMF‐induced histological alterations, including abnormalities, fatty degeneration, and inflammation in hepatocytes. Furthermore, HMF caused hepatotoxicity by negatively affecting the parameters related to oxidative stress. The results revealed that HMF elevated the expression levels of apoptotic genes in liver tissue and decreased the expression levels of antiapoptotic genes, thus promoting apoptosis. These results provide new evidence that HMF exerts its toxic effect on the liver through modulation of the Nrf2 signaling pathway and subsequent induction of oxidative stress and promotion of apoptotic processes.

## Graphical abstract

HMF may exert its toxic effect on the liver through regulation of the Nrf2 signaling pathway and subsequent induction of oxidative stress and promotion of apoptotic processes.

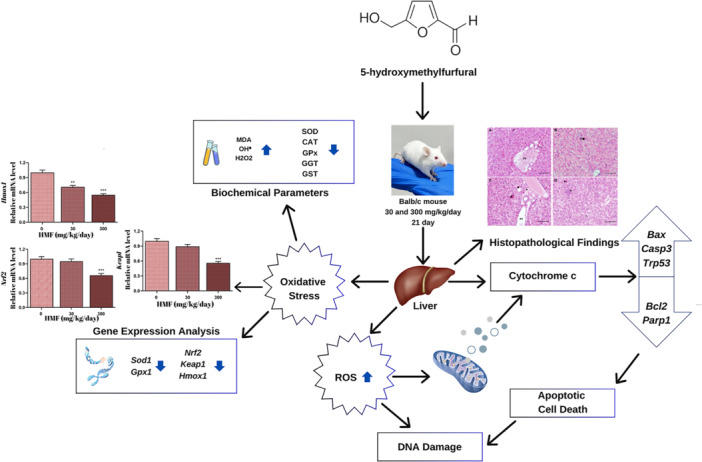

## 1 Introduction

5‐hydroxymethylfurfural (HMF) is an organic compound consisting of aromatic alcohol, aldehyde, and furan ring that is released during the heat treatment of carbohydrate‐rich foods. Research has established that HMF is an intermediate product formed during the degradation of hexose in acidic conditions or by the nonenzymatic Maillard reaction [1]. The World Health Organization and the European Union have performed food analysis to assess the prevalence of HMF in various food products [2]. According to the analysis, it has been reported that HMF is present in many foods, including coffee, honey, molasses, UHT milk, fruit and vegetable juices, breakfast cereals, and grains [3]. Daily HMF consumption varies between approximately 30–150 mg/kg per person, which is much higher than other heat exposure food contaminants such as furan and acrylamide [4]. HMF levels in coffee have been calculated as 110 mg/kg for traditionally roasted coffee, 625 mg/kg when sucrose is added before roasting, 1734 mg/kg in blended coffees, and 2480 mg/kg in soluble coffee [3]. Furthermore, honey includes significant levels of HMF because of the heat treatments used to make it marketable and the amount of sugar it contains, despite being a natural product that is used worldwide and has many stated health benefits [5].

Many foods that include sugar undergo heat treatment to produce HMF, which is readily absorbed through the gastrointestinal system after consumption and eliminated through urine following metabolism [6]. Moreover, the chemical industry makes extensive use of HMF, which has a furan ring [7]. Previous studies have demonstrated that at high doses (> 75 mg/kg), HMF exhibits a variety of toxicities, such as carcinogenicity, lung and kidney damage, and irritation of the skin and respiratory system [4]. The examination of biochemical parameters has revealed that HMF (200 mg/kg) causes kidney damage by increasing the levels of serum creatine and urea nitrogen. Additionally, the increase in alanine transaminase (ALT), aspartate transaminase (AST), and alkaline phosphatase (ALP) levels caused by HMF damages hepatocytes, myocardial cells, and skeletal tissue cells [8]. Another investigation demonstrated that high doses of HMF and 5‐sulphooxymethylfurfural (SMF), a minor metabolite of HMF, resulted in nephrotoxicity and hepatotoxicity in FVB/N mice [9]. An in vitro study utilizing GES‐1 gastric epithelial cells indicated that HMF induced apoptosis and resulted in oxidative stress, as evidenced by analyses of malondialdehyde (MDA), superoxide dismutase (SOD), catalase (CAT), and reactive oxygen species (ROS) [10]. The study conducted with zebrafish revealed that HMF significantly increased heart rate and pericardial edema rate, led to cardiac cell apoptosis, and caused defects in cardiovascular development. Also, it resulted in disorders of apoptosis‐related gene expression, elevated levels of ROS, and abnormal cardiovascular development in zebrafish larvae by inhibiting the Wnt signaling pathway [11]. A study involving albino mice was performed to examine the potential toxic effects of HMF on the liver. The findings suggested that HMF resulted in a notable reduction in albumin, globulin, and total protein levels, in addition to an increase in the activities of ALT, AST, and ALP. Furthermore, it was observed that HMF led to a reduction in SOD and CAT enzyme levels while increasing MDA levels [12].

A review of the literature reveals a limited number of studies addressing the potential hepatic toxicity of HMF [8, 9, 12, 13]. The liver plays a crucial role in metabolizing substances ingested into the body, converting toxic compounds into various derivatives for excretion. Research indicates that the majority of individuals with liver disease globally experience liver damage attributable to alcohol, in addition to exposure to chemicals and hazardous substances in diet [14]. Given that the liver is responsible for the metabolism of harmful chemicals, it is crucial to examine the consequences of HMF, a food contaminant. In this study, the role of HMF in liver toxicity was investigated by administering different concentrations of HMF (30 and 300 mg/kg) to adult male *Mus musculus* mice for 21 days. Liver tissues obtained from mice following HMF exposure were assessed using histological and histopathological methods. Furthermore, parameters of oxidative stress and the antioxidant defense system were analyzed to elucidate the damage induced by HMF in liver tissue. The mechanism of action of HMF in liver damage was investigated at the gene level for the first time by analyzing the expression changes of oxidative stress markers and apoptosis‐related genes.

## 2 Materials and Methods

### 2.1 Chemicals

HMF (purity ≥ 99%, Cas no.:67‐47‐0) was purchased from Sigma‐Aldrich (St. Louis, MO). We obtained other chemicals of the highest quality from standard commercial sources.

### 2.2 Animals and Experimental Design

In the present study, 7‐week‐old male Balb/c albino mice, with an average weight of 25–30 g, were obtained from the Aziz Sancar Experimental Medicine Institute, Department of Laboratory Animal Science (Istanbul, Turkey). Balb/c mice were housed in a controlled environment with a temperature of 22 ± 2°C, humidity of 55 ± 5%, and a consistent 12‐h light and 12‐h dark cycle. Mice were provided with unlimited access to water and food. Animal treatments were conducted with the approval of the Istanbul University Animal Experiments Local Ethics Committee (approval no. 2021/30). The EU directive 2010/63/EU on animal experimentation guided the conduct of the study. Reporting for the animal study followed ARRIVE guidelines. Animals were randomly divided into three groups, with seven animals in each group. The control group (group 1) received only normal saline (0.9% NaCl) by oral gavage for 21 days. HMF, dissolved in normal saline (0.9% NaCl), was administered to the experimental groups via gavage at doses of 30 mg/kg/day (group 2) and 300 mg/kg/day (group 3) for 21 days. The HMF doses administered (30 and 300 mg/kg/day) were established according to the amounts utilized in the 2010 National Toxicology Program research conducted in the USA [15]. Moreover, these doses have resulted in hepatic and renal impairment in prior in vivo investigations [9, 16]. The 21‐day exposure period applied in our study was determined based on previous toxicology studies to evaluate the subchronic effects of HMF. Several studies indicate that 21‐day short‐term exposure models are suitable and widely used to evaluate oxidative stress, inflammation, and cellular response mechanisms [12, 13, 17].

### 2.3 Tissues Preparation for Histological, Biochemical and Molecular Analysis

After 21 days of exposure, mice were anesthetized with ketamine hydrochloride (45 mg/kg) and then euthanized by cervical dislocation. At the end of euthanasia, liver tissues were appropriately collected for histological, biochemical, and molecular studies. Liver tissues were fixed in Bouin solution for histopathological evaluation. Liver tissue from each animal was promptly snap‐frozen on dry ice and thereafter stored in a −86°C freezer until biochemical and molecular analysis.

### 2.4 Histopathological Analysis

Liver tissues selected for histological examination were immersed in Bouin solution for 24 h to ensure adequate fixation. Paraffin blocks were produced according to normal tissue processing techniques for conventional morphological assessment. Sections of 5 μm in thickness were procured from each block and then stained with hematoxylin and eosin (H&E). Two slides were prepared for each mouse. These included three sections of each liver. Ten field regions were randomly chosen for each slice, and histopathological alterations were assessed using a light microscope. Counts of binucleated hepatocytes and nucleomegaly were analyzed at 40× magnification (Olympus IX71, Japan). Two people, unaware of group identification, conducted simultaneous analyses.

### 2.5 Measurement of Biomarkers

Liver tissues indicated for biochemical investigation were diluted with Tris‐HCl buffer (pH 7.4). The diluted tissues were homogenized using an IKA RW20 digital homogenizer. Following homogenization, the cell membranes were disrupted with an ultrasonicator and subsequently centrifuged in a refrigerated centrifuge. Supernatants obtained by centrifugation were analyzed for membrane lipid peroxidation, hydroxyl radicals (OH^●^), hydrogen peroxide (H_2_O_2_), total protein, superoxide dismutase (SOD), catalase (CAT), glutathione peroxidase (GPx), glutathione S‐transferase (GST), gamma glutamyl transpeptidase (GGT) and glutathione (GSH). The SMART BCA protein analysis kit was utilized for the measurement of total protein in liver tissue homogenates (AssayGenie, Dublin, Ireland). The protein sample concentrations were determined using the technique specified in the kit and by establishing a standard range from 0 to 2000 μg/ml for sample concentration measurement. The protein findings acquired were utilized in the computation of all biochemical analysis results.

The level of lipid peroxidation was assessed by measuring the malondialdehyde (MDA) concentration in the sample according to the method established by Heath and Parker [18]. The absorbance of the supernatant measured in the spectrophotometer was recorded against the blank at 532 nm. The method used to perform the detection of OH^●^ is that of Puntarulo and Cederbaum [19]. This method quantifies OH^●^ by the generation of formaldehyde, which then reacts with trichloroacetic acid, followed by spectrophotometric absorbance measurement at 570 nm. The method of Holland and Storey [20] was used to determine the amount of H_2_O_2_ that causes oxidative damage. The experimental procedure is based on the principle that the deviations increase as a result of the oxidation of acidified ferrocytochrome c, resulting in an enhanced absorbance at 550 nm. Our study employed the determination method for the SOD enzyme, which is critical to the enzymatic antioxidant defense system and was discovered by Marklund and Marklund [21]. This method involves the kinetic measurement of pyrogallol autoxidation by a SOD enzyme at 420 nm over 3 min, with data collected at 30‐second intervals. The method of Sinha [22] was used for the determination of the CAT enzyme in liver tissue. CAT enzyme determination was made by measuring the color change resulting from the reaction of H_2_O_2_ with dichromate/acetic acid spectrophotometrically at 570 nm. The Hafeman et al. [23] method was used as a basis for the demonstration of the GPx enzyme activity. The principle of this method determines the amount of enzyme based on the absorbance of the compound GPx and 5′,5‐ditiobis‐(2‐nitrobenzoik asit) form at 412 nm. GST determination was performed based on the method of Habig et al. [24], which is based on the colorimetric measurement of GSH‐ dinitrobenzene conjugate at a wavelength of 340 nm. GSH determination is based on the reaction of 5,5‐dithiobis‐(2‐nitrobenzoic acid) with GSH to measure glutathione reduced by 5‐thio‐nitrobenzoic acid at 412 nm [25].

### 2.6 Marker of Liver Function

The Orlowski and Meister [26] technique was used to illustrate the GGT enzyme. The tissue homogenates prepared for biochemical examination included 5 μmol l‐gamma‐glutamyl‐p‐nitroanilide, 10 μmol MgCl₂, 100 μmol Tris‐HCl (pH 9), and 1.5 N acetic acid. This technique involves the formation of free p‐nitroanilide from l‐gamma‐glutamyl‐p‐nitroanilide, which is then quantified using a spectrophotometer at a wavelength of 410 nm.

### 2.7 Quantitative Real‐Time PCR

Liver tissues of control and HMF (30 and 300 mg/kg/day) treated animals were first homogenized on ice to prepare for RNA isolation. Total RNA was extracted from liver tissue with the Total RNA Purification Mini Spin Kit (Genaxxon, Ulm, Germany) following the manufacturer's instructions. After determination of purity and quantity using the ND‐2000c NanoDrop device (Thermo Scientific, Darmstadt, Germany), the M‐MuLV First Strand cDNA Synthesis Kit (manufactured by Biomatic, Ontario, Canada) was used for cDNA synthesis. The primers synthesized for use in the study (provided from Integrated DNA Technologies, Leuven, Belgium) are presented in Table 1. The Light Cycler 480 device (Roche) was used to perform real‐time PCR reactions. Quantification of gene expression was performed using the LightCycler 480 SYBR Green 1 Master kit (Roche Applied Sciences, Mannheim, Germany) according to the methodology provided by the manufacturer. To normalize the relative quantification of mRNA levels, the housekeeping gene *Actb* was used as an internal control. Thermal cycling steps consisted of 95°C for 5 min, followed by 45 cycles of denaturation at 95°C for 10 s, primer annealing at the melting temperature (Tm) for 30 s, and extension at 72°C for 25 s. Data were analyzed using the 2^‐∆∆Ct^ approach developed by Livak and Schmittgen [27]. The resulting changes in gene expression levels were expressed as fold changes in HMF‐treated groups relative to control groups (*n* = 7).

**Table 1:** Primer sequences used for gene expression analyses.

Gene	Primer Sequence	NCBI gene ID
***Sod1***	Forward 5′­GTGATTGGGATTGCGCAGTA­3′ Reverse 5′­ TGGTTTGAGGGTAGCAGATGAGT ­3′	20655
***Gpx1***	Forward 5′­CGCTTTCGTACCATCGACATC­3′ Reverse 5′­GGGCCGCCTTAGGAGTTG­3′	14775
***Nrf2***	Forward 5′­CTTTAGTCAGCGACAGAAGGAC­3′ Reverse 5′­AGGCATCTTGTTTGGGAATGTG­3′	18024
***Hmox1***	Forward 5′­GTTTCCGCATACAACCAGTGA­3′ Reverse 5′­UUGGAUGUGUACCUCCUUGTT­3′	15368
***Keap1***	Forward 5′­CCCATGAGGCATCACCGTAG­3′ Reverse 5′­CATAGCCTCCGAGGACGTAG­3′	50868
***Parp1***	Forward 5′­CCATCGACGTCAACTACGAG­3′ Reverse 5′­GTGCGTGGTAGCATGAGTGT­3′	11545
***Bcl2***	Forward 5′­GACTGAGTACCTGAACCGG­3′ Reverse 5′­ATAGTTCCACAAAGGCATCC­3′	12043
***Bax***	Forward 5′­TGGCAGCTGACATGTTTTCTGAC­3′ Reverse 5′­CGTCCCAACCACCCTGGTCT­3′	12028
***Casp3***	Forward 5′­AATGGATTATCCTGAAATGGGC­3′ Reverse 5′­GAGCGAGATGACATTCCAG­3′	12367
***Trp53***	Forward 5′­AGAGACCGCCGTACAGAAGA­3′ Reverse 5′­GCATGGGCATCCTTTAACTC­3′	22059
***Actb***	Forward 5′­CGTTGACATCCGTAAAGAC­3′ Reverse 5′­TGGAAGGTGGACAGTGAG­3′	11461

Abbreviations: *Actb*, actin beta; *Bax*, BCL2‐associated X protein; *Bcl2*, B cell leukemia/lymphoma 2; *Casp3*, caspase 3, *Gpx1*, glutathione peroxidase 1; *Hmox1,* heme oxygenase 1; *Keap1*, kelch‐like ECH‐associated protein 1; *Nrf2*, nuclear factor erythroid derived 2; *Parp1*, poly (ADP‐ribose) polymerase family member 1; *Sod1*, superoxide dismutase 1; *Trp53*, transformation related protein 53,

### 2.8 Statistical Analysis

Statistical analysis of the data, comprising different doses administered to the experimental groups, was conducted using GraphPad Prism 9.0 (San Diego, CA, USA). The Shapiro‐Wilk normality test was used at first to assess the normal distribution of the acquired data. Subsequently, confirmation was obtained by one‐way analysis of variance (ANOVA), followed by Tukey's test for multiple group comparisons. The results were evaluated according to significance criteria of *p* < 0.05, *p* < 0.01, and *p* < 0.001. Tukey's test was employed for analyzing the changes in mRNA levels of genes compared to the applied concentration, utilizing the 2^‐ΔΔCt^ technique established by Livak and Schmittgen in 2001. *Actb* served as the reference gene, with significance graded as *p* < 0.05, *p* < 0.01, and *p* < 0.001 relative to the control group.

## 3 Results

### 3.1 Effects of HMF Administration on Liver Morphology

**Figure 1:**
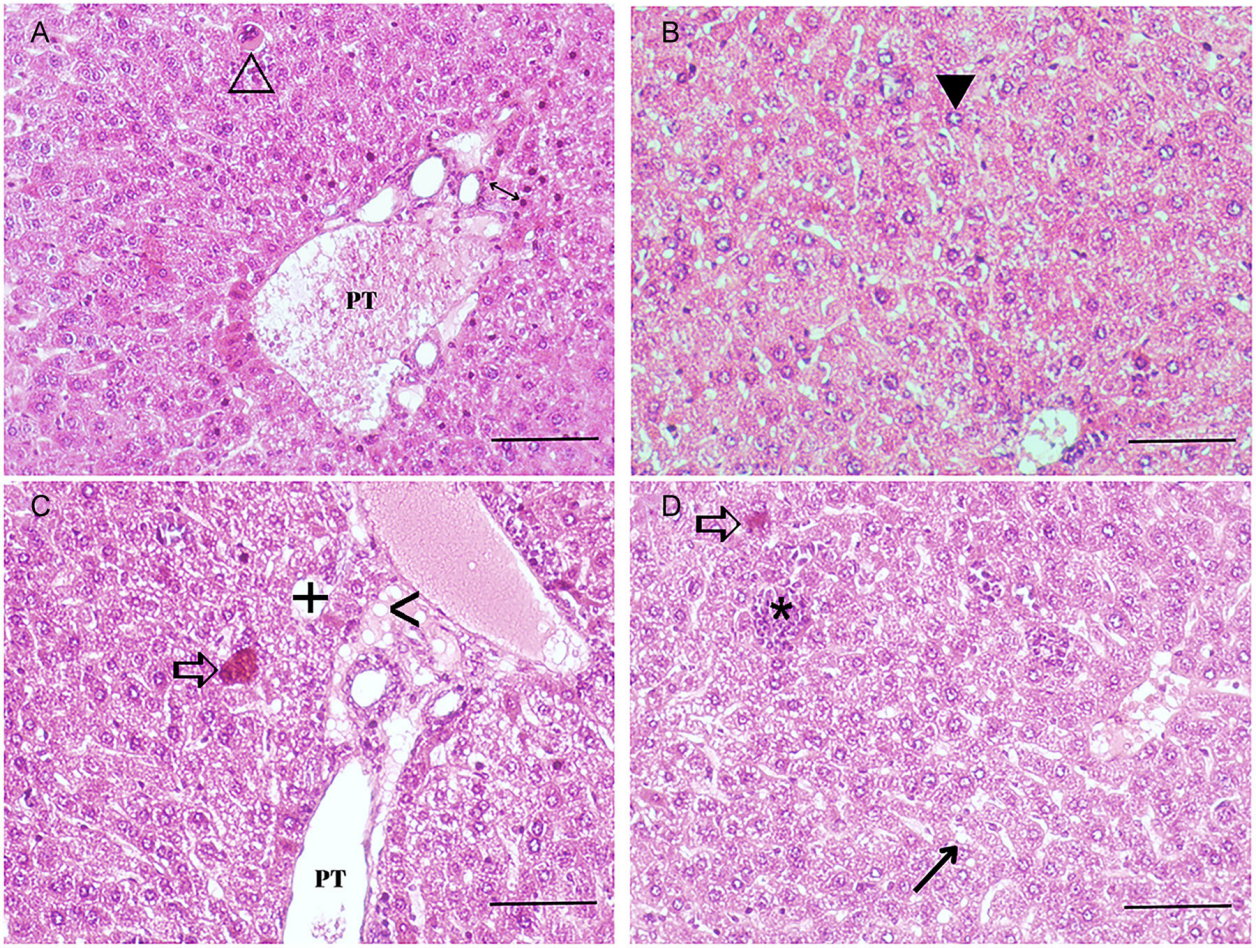
Pathological findings observed in liver tissues of mice (*n* = 7) with different concentrations of HMF. (A, B) HMF (30 mg/kg) group. (C, D) HMF (300 mg/kg) group. (H&E staining, magnification 100×).⊳: Necrosis, ▶: Vacuolization, ↔ Inflammatory hepatocytes, →: Sinusoidal dilatation, ⇨: Focal necrotic area, *: Area of inflammatory hepatocytes, **>**: Fat droplets, +: Perisinusoidal fibrosis. PT: portal triad.

**Figure 2:**
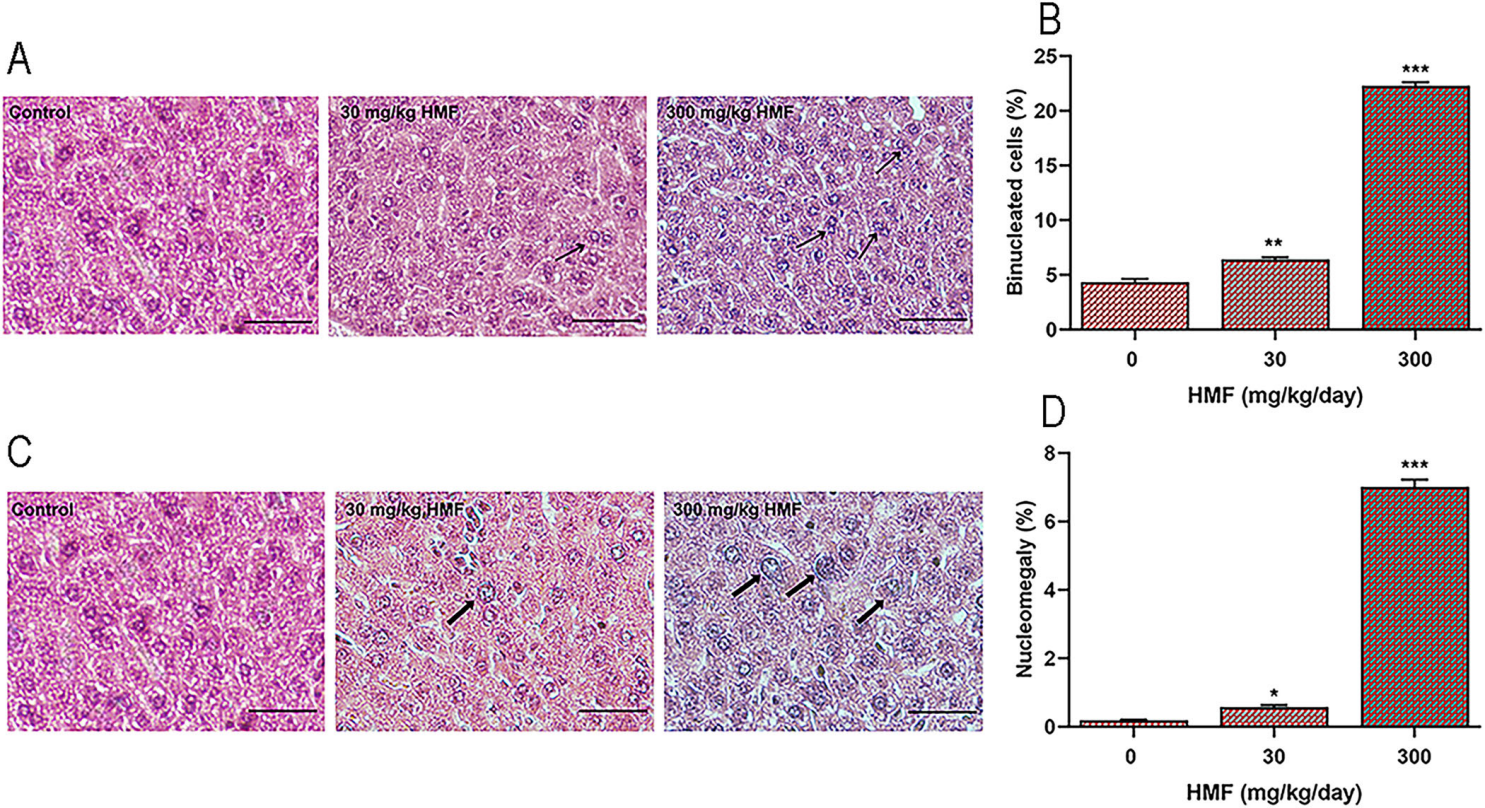
The frequency of hepatocytes with binuclei and nucleomegaly increased in the liver tissues of HMF‐treated mice (*n* = 7). (A) The figure displays a representative image of the binucleated hepatocytes in both the control and HMF groups. (B) The figure shows the percentage of binucleated hepatocytes calculated in the control and HMF groups. (C) The figure displays a representative image of hepatocytes with nucleomegaly in both the control and HMF groups. (D) The figure shows the percentage of hepatocytes with nucleomegaly calculated in the control and HMF groups. Values are expressed as mean ± SEM of seven mice. The experiments were repeated three times with similar results. *p < 0.05, ** *p* < 0.01, ***p < 0.001 compared with control. Thin black arrow: binucleated hepatocytes. Thick black arrow: nucleomegaly.

The general morphology of hepatocytes was found to be normal when the liver tissue sections in the control group stained with hematoxylin‐eosin were examined under a light microscope. It was discovered that the sinusoids were positioned normally inside the hepatocyte discs and had regular boundaries. It was found that the portal triad and central vein had consistent forms and locations. Figure 1 displays images of histological examinations of liver tissue sections from mice administered HMF. Analysis of the sections from individuals in the groups exposed to 30 and 300 mg/kg HMF doses for 21 days revealed degeneration in the general hepatocyte structure. Vacuolization occurred in hepatocytes in the HMF groups. Sinusoidal dilatation and mononuclear inflammation were noted in the 30 mg/kg HMF group. When liver tissues in the groups administered 300 mg/kg were compared to the 30 mg/kg HMF dose, increased sinusoidal dilatation, lymphocyte aggregation, degenerated portal area and central vein, and regional inflammation areas were observed. Figure 2 illustrates histological alterations, including binucleation (hepatocytes possessing two nuclei) and nucleomegaly (nuclear enlargement), detected in mouse liver tissue after HMF treatment. These morphological changes indicate structural deterioration of hepatocytes and toxic effects of HMF at the cellular level. Binucleation, linked to abnormalities in the cell division cycle, was seen to rise markedly in the HMF‐treated groups in a dose‐dependent manner (Figure 2A,B). Nucleomegaly, indicative of elevated metabolic activity or cellular stress, increased in a dose‐dependent manner in the HMF‐treated groups (Figure 2C,D). These parameters are among the early histological indicators of liver damage and are important in demonstrating the toxic effects of HMF.

### 3.2 Effects of HMF Administration on Oxidative Stress Markers and Antioxidant Enzyme Activities in Liver Tissue

Table 2 indicates significant elevations in the oxidative stress markers MDA, OH^●^, and H_2_O_2_ in the liver homogenates of mice following HMF treatment relative to the control group. The study evaluated the activities of SOD, CAT, and GPx enzymes in the antioxidant system and found that the activities of SOD and GPx significantly decreased only at 300 mg/kg/day of HMF compared to the control. On the other hand, the activity of the CAT enzyme was found to decrease significantly depending on the HMF dose. A significant decrease in GST activities was observed in the liver tissue of the HMF‐applied experimental groups compared to the control group for both doses of HMF. The experimental groups applied with 30 and 300 mg/kg HMF showed dramatic decreases in the activity of the nonenzymatic antioxidant GSH compared to the control.

**Table 2:** Analysis of oxidative stress indices in the liver of mice induced by HMF.

Parameters	Control	HMF (30 mg/kg/day)	HMF (300 mg/kg/day)
MDA (µmol/mg protein)	25.08 ± 2.8	30.08 ± 2.5*	43.33 ± 3.9***
OH^•^ (µmol/mg protein)	15.62 ± 1.2	27.93 ± 2.7**	31.07 ± 2.3***
H₂O₂ (µmol/mg protein)	1.61 ± 0.1	2.59 ± 0.2*	3.91 ± 0.2***
SOD (U/mg‐protein)	1.75 ± 0.1	1.68 ± 0.1	1.21 ± 0.1***
CAT (consumed nmol H_2_O_2_/min/mg protein)	6.34 ± 0.2	5.08 ± 0.1**	2.90 ± 0.1***
GPx (nmol glutathione consumed/mg protein)	11.97 ± 0.4	10.34 ± 0.5	6.59 ± 0.4***
GST (μmol CDNB‐GSH conjugate formed/min/mg protein)	2.01 ± 0.1	1.68 ± 0.1**	1.63 ± 0.1**
GSH (nM of GSH consumed/mg protein)	6.04 ± 0.3	4.39 ± 0.2***	3.95 ± 0.2***

*Note:* Values are expressed as mean ± SEM of seven mice. The experiments were repeated three times with similar results.

Abbreviations: CAT, catalase; CDNB, 1‐chloro‐2,4‐dinitrobenzene; GPx, glutathione peroxidase; GSH: glutathione; GST, glutathione S‐transferase; HMF, 5‐hydroxymethylfurfura; H₂O₂, hydrogen peroxide; MDA, malondialdehyde; OH•, hydroxyl radical; SOD: superoxide dismutase.

*p < 0.05, **p < 0.01, ***p < 0.001 compared with control.

### 3.3 Effects of HMF Administration on Liver Damage Indicator

**Figure 3:**
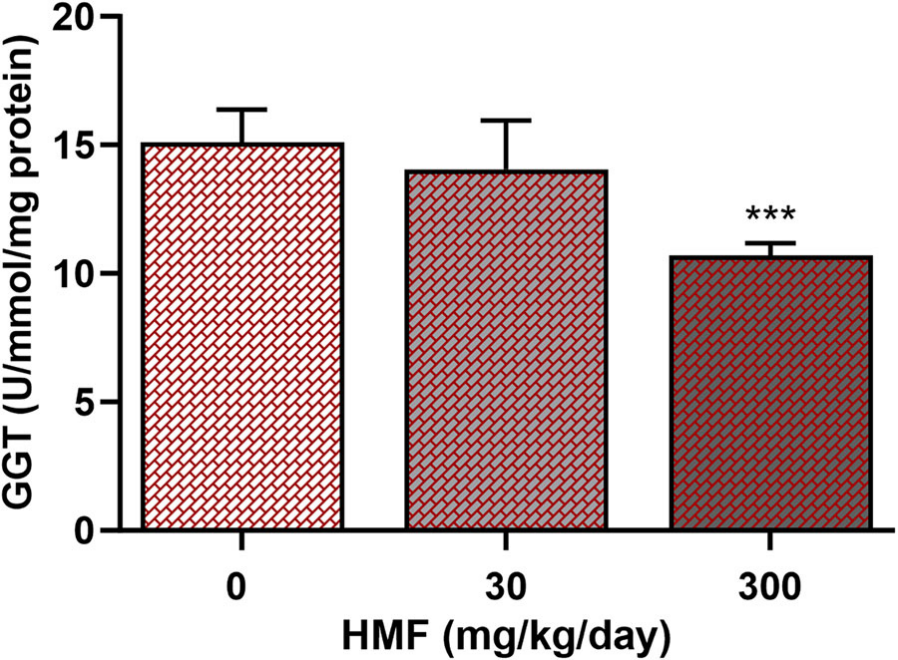
GGT enzyme activity in mouse liver tissues exposed to different doses of HMF. Values are expressed as mean ± SEM of seven mice. The experiments were repeated three times with similar results. ***p < 0.001 compared with control.

Figure 3 presents the effects of HMF on the GGT enzyme, which serves as an indicator of liver enzyme activities in mice. Exposure to HMF at a dose of 300 mg/kg resulted in a significant reduction of GGT enzyme activity (10.66 ± 0.2 U/mmol/mg protein) when compared to the control group (GGT: 15.11 ± 0.5 U/mmol/mg protein).

### 3.4 Effects of HMF Administration on the Expression Levels of Oxidative Stress‐Related Genes in Liver Tissue

**Figure 4:**
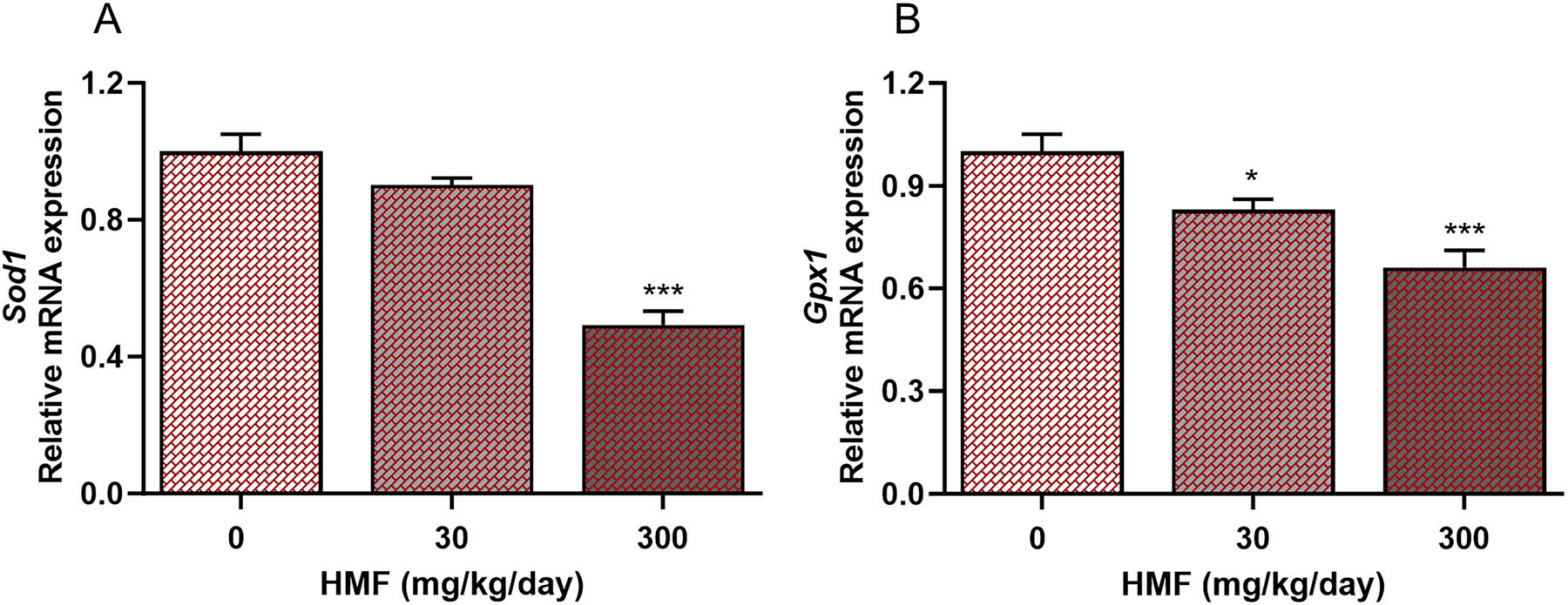
Expression of oxidative stress‐related genes in mouse liver tissues treated with HMF, conducted using RT‐PCR analysis. (A) *Sod1*, (B) *Gpx1*. Values are expressed as mean ± SEM of seven mice. The experiments were repeated three times with similar results. *p < 0.05, ***p < 0.001 compared with control. *Sod1*: superoxide dismutase 1, *Gpx1*: glutathione peroxidase 1.

Molecular analyses conducted on the liver tissue of mice receiving two distinct doses of HMF revealed a noticeable decrease in the expression level of the *Sod* gene when the 300 mg/kg HMF dose was compared to the control group (Figure 4A). A significant reduction in *Gpx* gene expression was observed in HMF‐treated groups relative to the control group (Figure 4B).

### 3.5 Effects of HMF Administration on the Expression Levels of Genes Involved in the Nrf2‐mediated Signaling Pathway in Liver Tissue

**Figure 5:**
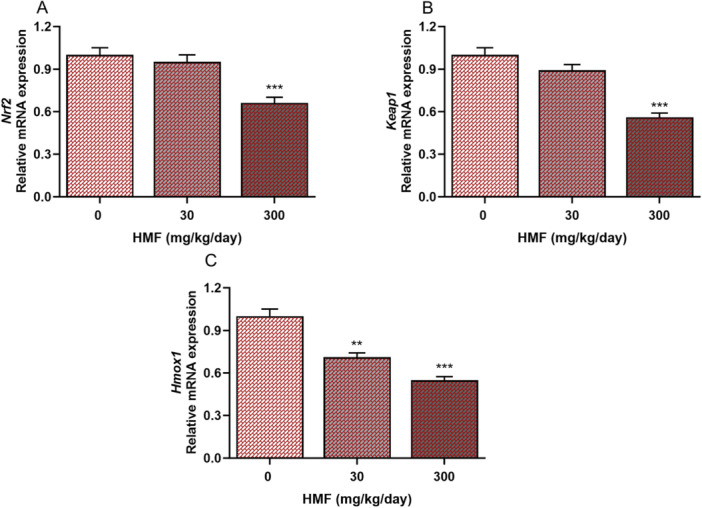
Expression of Nrf2 pathway‐related genes in mouse liver tissues treated with HMF, conducted using RT‐PCR analysis. (A) *Nrf2*, (B) *Keap1*, (C) *Hmox1*. Values are expressed as mean ± SEM of seven mice. The experiments were repeated three times with similar results. **p < 0.01, ***p < 0.001 compared with control. *Nrf2*: nuclear factor erythroid derived 2, *Keap1*: kelch‐like ECH‐associated protein 1, *Hmox1*: heme oxygenase 1.

Figure 5 illustrates the changes in the *Nrf2*, *Hmox1*, and *Keap1* gene expression levels in the liver tissues of mice exposed to HMF. It was determined that a 300 mg/kg HMF dose caused a significant decrease in *Nrf2* and *Keap1* mRNA levels compared to the control group (Figure 5A,B). Examining *Hmox1* levels revealed a significant decrease in HMF doses (Figure 5C).

### 3.6 Effects of HMF Administration on the Expression Levels of Mitochondrial Apoptosis Pathway‐Related Genes in Liver Tissue

**Figure 6:**
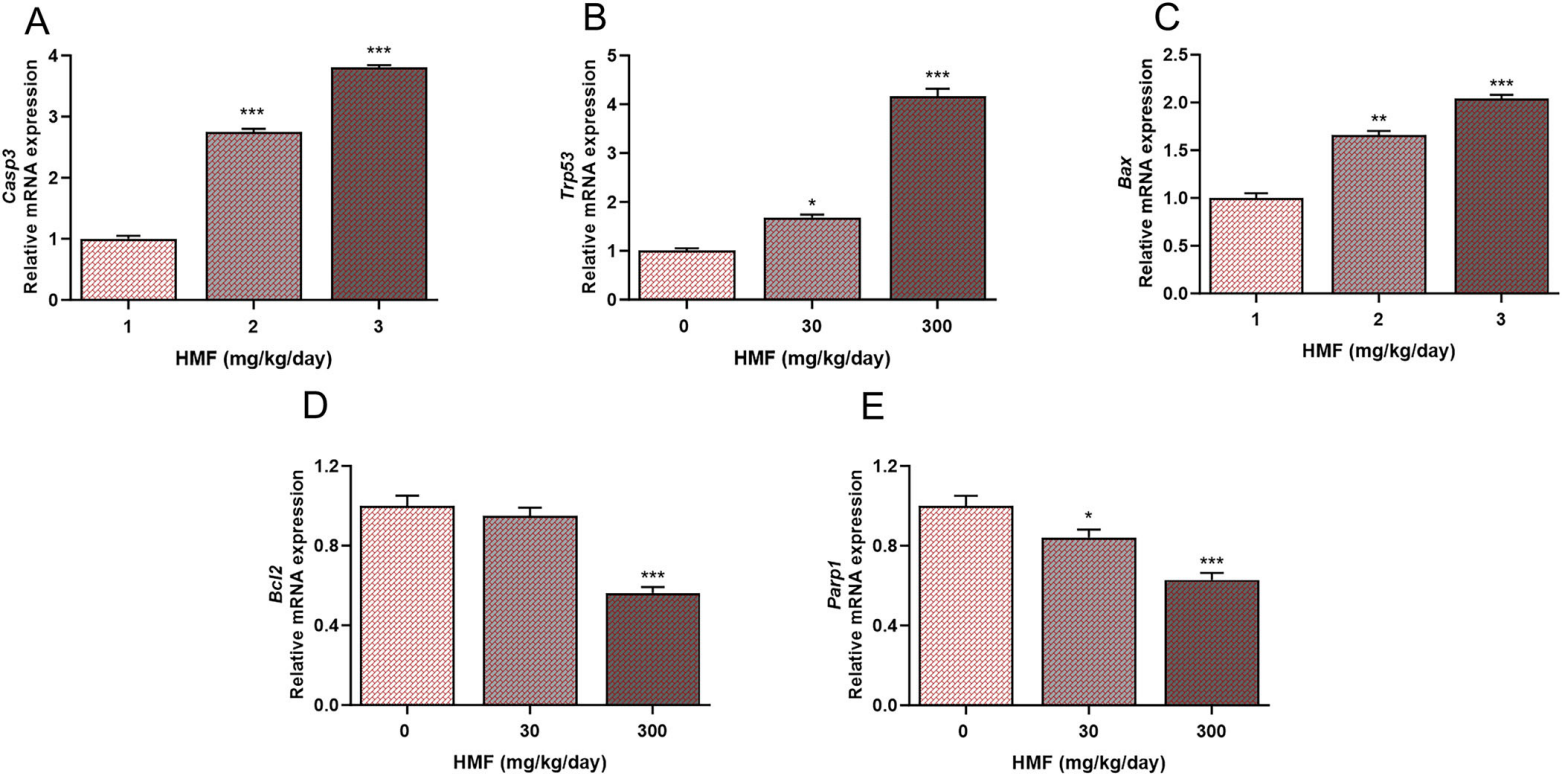
Expression of apoptotic pathway‐related genes in mouse liver tissues treated with HMF, conducted using RT‐PCR analysis. (A) *Casp3*, (B) *Trp53*, (C) *Bax*, (D) *Bcl2*, (E) *Parp1*. Values are expressed as mean ± SEM of seven mice. The experiments were repeated three times with similar results. *p < 0.05, **p < 0.01, ***p < 0.001 compared with control. *Casp3*: caspase 3, *Trp53*: transformation‐related protein 53, *Bcl2*: B‐cell leukemia/lymphoma 2, *Bax*: BCL2‐associated X protein, *Parp1*: poly (ADP‐ribose) polymerase family member 1.

The expression levels of apoptosis‐related genes were determined by qRT‐PCR (Figure 6). As shown in Figure 6A–C, HMF exposure was found to significantly increase the expression levels of *Casp3*, *Trp53*, and *Bax* genes in liver tissues. *Bcl2*, an antiapoptotic gene, was significantly downregulated in HMF‐treated groups compared to control (Figure 6D). HMF exposure significantly decreased the expression level of *Parp1*, which is associated with caspase‐dependent apoptosis (Figure 6E).

## 4 Discussion

Both in vivo and in vitro studies have been conducted to evaluate the negative effects of HMF on human health [1, 12, 28]. Studies have established that HMF induces oxidative stress by increasing ROS and causes genotoxicity and neurotoxicity [1, 29]. Moreover, a limited number of studies indicate that HMF induces apoptosis due to DNA damage [10, 30]. The liver is responsible for the biotransformation of harmful chemicals and mitigation of their toxic effects in the body. This study examined the response of the liver to HMF exposure. In this context, it was aimed to elucidate the mechanism of action of HMF in the liver by examining histopathological analysis, oxidative stress and antioxidant system parameters, and the expression levels of oxidative stress and apoptosis‐related genes in the liver tissues of mice exposed to 30 and 300 mg/kg doses of HMF for 21 days. Although the doses used in this study appear higher than the average daily HMF exposure levels through diet (approximately 30–150 mg/day), interspecies dose conversion calculations based on body surface area indicate that these values correspond to approximately 5–50 mg/kg/day in humans [31]. Frequent consumption of HMF‐rich foods such as coffee, honey, dried fruits, and caramelized products may increase the risk of chronic and cumulative exposure in humans. Therefore, subchronic experimental models using higher doses are considered relevant for evaluating the long‐term hepatotoxic effects of HMF. The findings of this study reveal the mechanistic toxicity that may occur in the liver as a result of high and prolonged HMF exposure.

In our study, histopathological examinations revealed that portal triad deformation, enlarged sinusoidal networks, lymphocyte aggregation, inflammation, and fatty degeneration occurred in hepatocytes after exposure to HMF in liver tissue. In a study conducted in line with our research, it was shown that histopathological changes occurred in liver tissue as a result of HMF exposure at a dose of 250 mg/kg [12]. Another study showed that long‐term exposure to HMF at 188 and 375 mg/kg and higher doses caused adenomas in the liver tissues of mice [15]. Research showing 100 mg/kg of HMF to mice for 22 days revealed the presence of edema in liver histological sections [13]. When the findings were compared with the literature, it was determined that HMF caused liver damage by causing necrotic hepatocytes, binucleate, and nucleomegalic hepatocytes even at a very low dose of 30 mg/kg.

The balance between oxidative stress and the antioxidant defense system is disrupted after exposure to hazardous substances, resulting in elevated lipid peroxidation due to increased ROS levels [32]. Increases in H_2_O_2_ and OH^●^ levels and changes in the amount of MDA, the end product of lipid peroxidation, are used as indicators of cell damage [33]. A study indicated that a 250 mg/kg dose of HMF administered to mice resulted in a significant increase in MDA levels in liver tissue [12]. The study using rats assessed MDA levels in several tissues following HMF administration, revealing that a dose of 100 mg/kg of HMF significantly elevated MDA levels in the liver [34]. In parallel with the limited studies mentioned above, this study found that HMF (30 and 300 mg/kg) caused significant increases in lipid peroxidation in the liver tissues of mice. It was also shown that HMF caused oxidative stress by raising the levels of H_2_O_2_ and OH^●^ radicals, which was investigated for the first time in liver tissue.

Enzymatic and nonenzymatic antioxidants, such as CAT, SOD, GPx, GSH, and GST, are key components of the antioxidant defense system that mitigate oxidative damage within cells [35]. A study found that a dose of 250 mg/kg of HMF significantly reduced the activities of SOD, CAT, and GSH in mouse liver [12]. In another study, biochemical analyses of liver tissue demonstrated that a 100 mg/kg HMF dose administered to rats significantly decreased GST, SOD, and GSH activities [34]. Due to the limited studies focusing on antioxidant system parameters of HMF in liver tissue, the interaction mechanism of HMF with the antioxidant system has not been fully elucidated. In this context, the HMF‐induced antioxidant system parameters in the liver, which is the organ responsible for minimizing the effects of food contaminants, were evaluated by biochemical and molecular methods. The study demonstrated that HMF significantly reduces the enzyme activity and mRNA expression levels of SOD and GPx in liver tissue. Also, substantial reductions in CAT, GST, and GSH activities disturbed the oxidant/antioxidant equilibrium, demonstrating that HMF is a chemical that markedly induces oxidative damage in hepatic tissue. GGT is an enzyme commonly used as a biomarker in liver dysfunctions and plays an important role in oxidative stress by breaking down extracellular radicals [36]. There is no study evaluating the activity of the GGT enzyme, which is known to be present at high levels in liver tissue, after HMF exposure. According to the literature, it has been found that the activity of the GGT enzyme in the serum of mice or rats significantly increases due to toxicity when exposed to different toxic compounds [37, 38]. Our investigation revealed a significant decline in GGT enzyme activity at a dose of 300 mg/kg of HMF. The decrease in GGT enzyme activity measured in liver tissue homogenates is explained by the increase in the number of apoptotic and necrotic hepatocytes after HMF exposure [39].

The expression levels of genes that regulate oxidative stress induction or suppression alter due to exposure to toxic substances. During oxidative stress, Nrf‐2 dissociates from Keap‐1 and translocates to the nucleus, where it initiates a response to oxidative damage by increasing HO‐1 expression, hence mitigating ROS generation [40]. Moreover, HO‐1 is recognized for its role in regulating apoptosis and modulating inflammation [41]. A study using rat liver showed that acrylamide, a food contaminant, induces liver damage by interfering with the expression of the *Nrf2* and *Keap1* genes [42]. Another investigation demonstrated that acrolein induced lung diseases via elevating the expression of the *Hmox1* gene [43]. The present study examined the involvement of the Nrf2/Keap1/HO‐1 signaling pathway in HMF‐induced hepatic injury for the first time. The expression levels of *Keap1*, *Hmox1*, and *Nrf2* genes were considerably reduced following exposure to HMF. This result elucidates the mechanism of action of oxidative damage caused by HMF, highlighting its role in liver dysfunction.

Toxic substances cause disruption of tissue homeostasis by targeting genes that induce and suppress apoptosis [44]. In a study conducted to investigate the role of HMF in the apoptosis mechanism, apoptotic changes were investigated by exposing gastric mucosal epithelial cells to the lowest 4 and highest 16 mM concentrations of HMF. The results showed that HMF reduced *Bcl2* expression and promoted apoptosis by inducing *Bax* [10]. Another study reported that HMF induces apoptosis by reducing *Bcl2* mRNA expression [12]. HMF triggered apoptosis by enhancing caspase activation in direct correlation with the dose‐dependent rise in the A375 melanoma cell line. In addition, it has been determined that HMF induces apoptosis by causing both caspase‐3 stimulation of the *Parp1* gene and ROS‐induced DNA damage [45]. This study demonstrated that HMF leads to apoptosis by elevating the expression levels of *Bax*, *Casp3*, and *Trp53* while decreasing the expression levels of *Parp1* and *Bcl2* in liver tissue.

In our study, the main molecular mechanisms of the toxic effects of HMF on the liver include the suppression of the Nrf2/Keap1/HO‐1 signaling pathway, which is an important component of the cellular antioxidant system. Inhibition of Nrf2 disrupts the intracellular oxidative balance and causes the accumulation of ROS, leading to increased oxidative stress [46]. This scenario leads to the disturbance of mitochondrial membrane potential and the initiation of apoptotic signaling pathways [47]. The increase in *Casp3*, *Bax*, and *Trp53* gene expression and the decrease in *Bcl2* and *Parp1* levels observed in our study suggest that HMF has a proapoptotic effect targeting the mitochondrial pathway. These findings are consistent with studies in the molecular toxicology literature, demonstrating that ROS production, lysosomal dysfunction, inflammation, and alternative forms of cell death (e.g., ferroptosis, pyroptosis) contribute to the modulation of toxic responses [46, 48, 49, 50]. In this respect, the study provides important contributions to the molecular toxicology literature by revealing that the hepatotoxic effects of HMF are not only histological but also disruptions in intracellular molecular regulatory networks.

This study provides important mechanistic data by examining the toxic effects of HMF on the liver in detail at both histological and molecular levels. However, some limitations should be considered. The use of only two doses in the study limited the detailed evaluation of the dose–response relationship. In addition, although the 21‐day subchronic exposure period is consistent with the literature, it does not provide information on longer‐term toxicity and recovery processes. The use of only male BALB/c mice excluded biological differences related to gender and genetic background. Molecular analyses were limited to the gene level related to the Nrf2 signaling pathway and apoptosis; protein expression, functional activities (e.g., nuclear translocation), and cellular apoptosis indicators (e.g., TUNEL staining) were not evaluated. Furthermore, considering the biotransformation differences between species, caution should be exercised in interpreting the results for humans. These limitations indicate the need for more comprehensive approaches and translational validations in future studies.

## 5 Conclusions

This study presents novel data that elucidate the mechanism of action of HMF on hepatic injury. Our current study proves that HMF triggers oxidative stress and apoptosis by activating the Nrf2/Keap1/HO‐1 signaling pathway in the liver. The histopathological findings indicate that HMF induces sinusoidal dilatation, lymphocyte aggregation, degeneration of the portal area and central vein, localized inflammation, and an increase in the number of hepatocytes presenting binucleation and nucleomegaly in liver tissue. Moreover, significant elevations in HMF‐induced oxidative stress markers and considerable decreases in antioxidant enzyme activity were observed, leading to an imbalance in the cellular antioxidant/oxidant equilibrium. The alterations in the expression levels of apoptotic genes also confirmed that HMF causes hepatotoxicity through multiple pathways. Our research may provide a theoretical basis for further investigating the health risks triggered by hazardous heat‐related food contaminants.

## Author Contributions

**Hatice Kurtel:** investigation, methodology. **Yasemin Aydin:** conceptualization, investigation, formal analysis, visualization, writing – review and editing. **Banu Orta‐Yilmaz:** Project administration, funding acquisition, supervision, conceptualization, methodology, investigation, formal analysis, visualization, writing – original draft, writing – review and editing.

## Conflicts of Interest

The authors declare no conflicts of interest.

## Data Availability

The data that support the findings of this study are available from the corresponding author upon reasonable request.

## References

[1] L. J. K. Durling , L. Busk , and B. E. Hellman , “Evaluation of the DNA Damaging Effect of the Heat‐Induced Food Toxicant 5‐Hydroxymethylfurfural (HMF) in Various Cell Lines With Different Activities of Sulfotransferases,” Food and Chemical Toxicology 47, no. 4 (2009): 880–884, 10.1016/j.fct.2009.01.022. 19271322

[2] E. M. S. M. Gaspar and J. F. Lopes , “Simple Gas Chromatographic Method for Furfural Analysis,” Journal of Chromatography A 1216, no. 14 (2009): 2762–2767, 10.1016/j.chroma.2008.10.049. 18976770

[3] A. Choudhary , V. Kumar , S. Kumar , I. Majid , P. Aggarwal , and S. Suri , “5‐Hydroxymethylfurfural (HMF) Formation, Occurrence and Potential Health Concerns: Recent Developments,” Toxin Reviews 40, no. 4 (2021): 545–561, 10.1080/15569543.2020.1756857.

[4] A. A. Rosatella , S. P. Simeonov , R. F. M. Frade , and C. A. M. Afonso , “5‐Hydroxymethylfurfural (HMF) as a Building Block Platform: Biological Properties, Synthesis and Synthetic Applications,” Green Chemistry 13, no. 4 (2011): 754–793, 10.1039/c0gc00401d.

[5] U. M. Shapla , M. Solayman , N. Alam , M. I. Khalil , and S. H. Gan , “5‐Hydroxymethylfurfural (HMF) Levels in Honey and Other Food Products: Effects on Bees and Human Health,” Chemistry Central Journal 12, no. 1 (2018): 35, 10.1186/s13065-018-0408-3. 29619623 PMC5884753

[6] M. Anese , L. Manzocco , S. Calligaris , and M. C. Nicoli , “Industrially Applicable Strategies for Mitigating Acrylamide, Furan, and 5‐Hydroxymethylfurfural in Food,” Journal of Agricultural and Food Chemistry 61, no. 43 (2013): 10209–10214, 10.1021/jf305085r. 23627283

[7] F. A. Kucherov , L. V. Romashov , K. I. Galkin , and V. P. Ananikov , “Chemical Transformations of Biomass‐Derived C6‐Furanic Platform Chemicals for Sustainable Energy Research, Materials Science, and Synthetic Building Blocks,” ACS Sustainable Chemistry & Engineering 6, no. 7 (2018): 8064–8092, 10.1021/acssuschemeng.8b00971.

[8] D. Özkök and S. Silici , “Effects of Honey HMF on Enzyme Activities and Serum Biochemical Parameters of Wistar Rats,” Environmental Science and Pollution Research 23, no. 20 (2016): 20186–20193, 10.1007/s11356-016-7218-8. 27439754

[9] M. Bauer‐Marinovic , F. Taugner , S. Florian , and H. Glatt , “Toxicity Studies With 5‐Hydroxymethylfurfural and Its Metabolite 5‐Sulphooxymethylfurfural in Wild‐Type Mice and Transgenic Mice Expressing Human Sulphotransferases 1A1 and 1A2,” Archives of Toxicology 86, no. 5 (2012): 701–711, 10.1007/s00204-012-0807-5. 22349055

[10] Y. Qiu , X. Lin , Z. Chen , B. Li , and Y. Zhang , “5‐Hydroxymethylfurfural Exerts Negative Effects on Gastric Mucosal Epithelial Cells by Inducing Oxidative Stress, Apoptosis, and Tight Junction Disruption,” Journal of Agricultural and Food Chemistry 70, no. 12 (2022): 3852–3861, 10.1021/acs.jafc.2c00269. 35311281

[11] Y. Jiang , N. Geng , M. Wang , W. Wu , N. Feng , and X. Zhang , “5‐HMF Affects Cardiovascular Development in Zebrafish Larvae via Reactive Oxygen Species and Wnt Signaling Pathways,” Comparative Biochemistry and Physiology Part C: Toxicology & Pharmacology 262 (2022): 109452, 10.1016/S1875-5364(15)30095-9. 36067963

[12] K. M. El Bohi , M. H. Ghoniem , H. H. Azab , H. Ali , and M. R. Farag , “Extra Virgin Olive Oil Enhances the Hepatic Antioxidant Defense and Inhibits Cytogenotoxic Effects Evoked by 5‐Hydroxymethylfurfural in Mice,” Environmental Science and Pollution Research 27, no. 11 (2020): 11882–11891, 10.1007/s11356-020-07659-x. 31981028

[13] L. Fan , F. Wang , Q. Yao , et al., “Lactoferrin Could Alleviate Liver Injury Caused by Maillard Reaction Products With Furan Ring Through Regulating Necroptosis Pathway,” Food Science & Nutrition 9, no. 7 (May 2021): 3449–3459, 10.1002/fsn3.2254. 34262705 PMC8269604

[14] A. Brooks , X. Liang , Y. Zhang , et al., “Liver Organoid as a 3D In Vitro Model for Drug Validation and Toxicity Assessment,” Pharmacological Research 169 (2021): 105608, 10.1016/j.phrs.2021.105608. 33852961

[15] National Toxicology Program. NTP Toxicology and Carcinogenesis Studies of 5‐(Hydroxymethyl)‐2‐furfural (CAS No. 67‐47‐0) in F344/N Rats and B6C3F1 Mice (Gavage Studies),” National Toxicology Program Technical Report Series 554 (2010): 7. 20725154

[16] C. Svendsen , T. Husøy , H. Glatt , J. E. Paulsen , and J. Alexander , “5‐Hydroxymethylfurfural and 5‐Sulfooxymethylfurfural Increase Adenoma and Flat ACF Number in the Intestine of Min/+ Mice,” Anticancer Research 29, no. 6 (2009): 1921–1926. 19528448

[17] X. Guo , L. Weng , L. Yi , and D. Geng , “Toxicological Safety Evaluation in Acute and 21‐Day Studies of Ethanol Extract From *Solanum lyratum* Thunb,” Evidence‐Based Complementary and Alternative Medicine 2022 (March 2022): 8518324, 10.1155/2022/8518324. 35399634 PMC8991412

[18] R. L. Heath and L. Packer , “Photoperoxidation in Isolated Chloroplasts,” Archives of Biochemistry and Biophysics 125, no. 1 (1968): 189–198, 10.1016/0003-9861(68)90654-1. 5655425

[19] S. Puntarulo and A. I. Cederbaum , “Effect of Oxygen Concentration on Microsomal Oxidation of Ethanol and Generation of Oxygen Radicals,” Biochemical Journal 251, no. 3 (1988): 787–794, 10.1042/bj2510787. 3415646 PMC1149072

[20] M. K. Holland and B. T. Storey , “Oxygen Metabolism of Mammalian Spermatozoa. Generation of Hydrogen Peroxide by Rabbit Epididymal Spermatozoa,” Biochemical Journal 198, no. 2 (1981): 273–280, 10.1042/bj1980273. 7326006 PMC1163245

[21] S. Marklund and G. Marklund , “Involvement of the Superoxide Anion Radical in the Autoxidation of Pyrogallol and a Convenient Assay for Superoxide Dismutase,” European Journal of Biochemistry 47, no. 3 (1974): 469–474, 10.1111/j.1432-1033.1974.tb03714.x. 4215654

[22] A. K. Sinha , “Colorimetric Assay of Catalase,” Analytical Biochemistry 47, no. 2 (1972): 389–394, 10.1016/0003-2697(72)90132-7. 4556490

[23] D. G. Hafeman , R. A. Sunde , and W. G. Hoekstra , “Effect of Dietary Selenium on Erythrocyte and Liver Glutathione Peroxidase in the Rat,” Journal of Nutrition 104, no. 5 (1974): 580–587, 10.1093/jn/104.5.580. 4823943

[24] W. H. Habig , M. J. Pabst , and W. B. Jakoby , “Glutathione S‐Transferases,” Journal of Biological Chemistry 249, no. 22 (1974): 7130–7139. 4436300

[25] G. L. Ellman , “Tissue Sulfhydryl Groups,” Archives of Biochemistry and Biophysics 82, no. 1 (1959): 70–77, 10.1016/0003-9861(59)90090-6. 13650640

[26] M. Orlowski and A. Meister , “Isolation of γ‐Glutamyl Transpeptidase From Hog Kidney,” Journal of Biological Chemistry 240, no. 1 (1965): 338–347. 14253434

[27] K. J. Livak and T. D. Schmittgen , “Analysis of Relative Gene Expression Data Using Real‐Time Quantitative PCR and the 2−ΔΔCT Method,” Methods 25, no. 4 (2001): 402–408, 10.1006/meth.2001.1262. 11846609

[28] S. Elmaoğulları , E. Kadan , E. Anadol , et al., “Effects of 5‐Hydroxymethylfurfural on Pubertal Development of Female Wistar Rats,” Journal of Clinical Research in Pediatric Endocrinology 12, no. 1 (2020): 79–85, 10.4274/jcrpe.galenos.2019.2019.0080. 31475510 PMC7127893

[29] I. Severin , C. Dumont , A. Jondeau‐Cabaton , V. Graillot , and M. C. Chagnon , “Genotoxic Activities of the Food Contaminant 5‐Hydroxymethylfurfural Using Different In Vitro Bioassays,” Toxicology Letters 192, no. 2 (2010): 189–194, 10.1016/j.toxlet.2009.10.022. 19879342

[30] Z. Q. Jiang , Y. X. Ma , M. H. Li , X. Q. Zhan , X. Zhang , and M. Y. Wang , “5‐Hydroxymethylfurfural Protects Against ER Stress‐Induced Apoptosis in GalN/TNF‐α‐injured L02 Hepatocytes Through Regulating the PERK‐eIF2α Signaling Pathway,” Chinese Journal of Natural Medicines 13, no. 12 (2015): 896–905, 10.1016/S1875-5364(15)30095-9. 26721708

[31] S. Reagan‐Shaw , M. Nihal , and N. Ahmad , “Dose Translation From Animal to Human Studies Revisited,” FASEB Journal 22, no. 3 (2008): 659–661, 10.1096/fj.07-9574LSF. 17942826

[32] E. Zamani , M. Shokrzadeh , M. Fallah , and F. Shaki , “A Review of Acrylamide Toxicity and Its Mechanism,” Pharmaceutical and Biomedical Research 3, no. 1 (2017): 1–7, 10.18869/acadpub.pbr.3.1.1.

[33] A. Pompella , E. Maellaro , A. F. Casini , M. Ferrali , L. Ciccoli , and M. Comporti , “Measurement of Lipid Peroxidation In Vivo: A Comparison of Different Procedures,” Lipids 22, no. 3 (1987): 206–211, 10.1007/BF02537304. 3574001

[34] Q. Zhao , J. Ou , C. Huang , et al., “Absorption of 1‐Dicysteinethioacetal‐5‐Hydroxymethylfurfural in Rats and Its Effect on Oxidative Stress and Gut Microbiota,” Journal of Agricultural and Food Chemistry 66, no. 43 (2018): 11451–11458, 10.1021/acs.jafc.8b04260. 30303013

[35] B. B. Mathew , A. Tiwari , and S. K. Jatawa , “Free Radicals and Antioxidants: A Review,” Journal of Pharmaceutical Research 4, no. 12 (2011): 4340–4343.

[36] J. B. Whitfield , “Gamma Glutamyl Transferase,” Critical Reviews in Clinical Laboratory Sciences 38, no. 4 (2001): 263–355, 10.1080/20014091084227. 11563810

[37] D. Gupta , S. Shrivastava , S. S. Gupte , and S. Shukla , “Caffeic Acid Attenuates Acrylamide Induced Biochemical, Hematological, and Histological Alterations in Rats,” Pharmacological Research 3 (2024): 100031, 10.1016/j.prenap.2024.100031.

[38] S. A. Rajput , A. Shaukat , K. Wu , et al., “Luteolin Alleviates AflatoxinB1‐Induced Apoptosis and Oxidative Stress in the Liver of Mice Through Activation of Nrf2 Signaling Pathway,” Antioxidants 10, no. 8 (2021): 1268, 10.3390/antiox10081268. 34439516 PMC8389199

[39] F. Bulle , P. Mavier , E. S. Zafrani , et al., “Mechanism of γ–Glutamyl Transpeptidase Release in Serum During Intrahepatic and Extrahepatic Cholestasis in the Rat: A Histochemical, Biochemical and Molecular Approach,” Hepatology 11, no. 4 (1990): 545–550, 10.1002/hep.1840110404. 1970323

[40] F. Kong , B. H. Lee , and K. Wei , “5‐Hydroxymethylfurfural Mitigates Lipopolysaccharide‐Stimulated Inflammation via Suppression of MAPK, NF‐κB and mTOR Activation in RAW 264.7 Cells,” Molecules 24, no. 2 (January 2019): 275, 10.3390/molecules24020275. 30642099 PMC6359491

[41] A. Loboda , M. Damulewicz , E. Pyza , A. Jozkowicz , and J. Dulak , “Role of Nrf2/HO‐1 System in Development, Oxidative Stress Response and Diseases: An Evolutionarily Conserved Mechanism,” Cellular and Molecular Life Sciences 73, no. 17 (2016): 3221–3247, 10.1007/s00018-016-2223-0. 27100828 PMC4967105

[42] D. B. Donmez , S. Kacar , R. Bagci , and V. Sahinturk , “Protective Effect of Carnosic Acid on Acrylamide‐Induced Liver Toxicity in Rats: Mechanistic Approach Over Nrf2‐Keap1 Pathway,” Journal of Biochemical and Molecular Toxicology 34, no. 9 (2020): e22524, 10.1002/jbt.22524. 32383547

[43] R. Xiong , Q. Wu , L. Muskhelishvili , et al., “Evaluating Mode of Action of Acrolein Toxicity in an In Vitro Human Airway Tissue Model,” Toxicological Sciences 166, no. 2 (2018): 451–464, 10.1093/toxsci/kfy226. 30204913

[44] P. M. Henson , D. L. Bratton , and V. A. Fadok , “Apoptotic Cell Removal,” Current Biology 11, no. 19 (2001): R795–R805, 10.1016/s0960-9822(01)00474-2. 11591341

[45] L. Zhao , J. Su , L. Li , et al., “Mechanistic Elucidation of Apoptosis and Cell Cycle Arrest Induced by 5‐Hydroxymethylfurfural, the Important Role of ROS‐Mediated Signaling Pathways,” Food Research International 66 (2014): 186–196, 10.1016/j.foodres.2014.08.051.

[46] D. Wang , K. Yin , Y. Zhang , et al., “Novel Pathways of Fluoride‐Induced Hepatotoxicity: P53‐Dependent Ferroptosis Induced by the SIRT1/Foxos Pathway and Nrf2/HO‐1 Pathway,” Comparative Biochemistry and Physiology Part C: Toxicology & Pharmacology 264 (2023): 109526, 10.1016/j.cbpc.2022.109526. 36455829

[47] J. Liu , H. Zhao , Y. Wang , Y. Shao , J. Li , and M. Xing , “Alterations of Antioxidant Indexes and Inflammatory Cytokine Expression Aggravated Hepatocellular Apoptosis Through Mitochondrial and Death Receptor‐Dependent Pathways in *Gallus gallus* Exposed to Arsenic and Copper,” Environmental Science and Pollution Research 25, no. 16 (2018): 15462–15473, 10.1007/s11356-018-1757-0. 29569195

[48] X. Shi , T. Xu , M. Gao , et al., “Combined Exposure of Emamectin Benzoate and Microplastics Induces Tight Junction Disorder, Immune Disorder and Inflammation in Carp Midgut via Lysosome/ROS/Ferroptosis Pathway,” Water Research 257 (2024): 121660, 10.1016/j.watres.2024.121660. 38688190

[49] T. Xu , T. Chen , X. Shi , J. Ding , S. Chen , and H. Lin , “Co‐Exposure of Bisphenol A and Selenium Deficiency Induces Pyroptosis via ROS/NLRP3 Pathway in Chicken Spleen,” Poultry Science 103, no. 10 (2024): 104150, 10.1016/j.psj.2024.104150. PMC11379661 39146921

[50] K. Yin , D. Wang , Y. Zhang , et al., “Polystyrene Microplastics Promote Liver Inflammation by Inducing the Formation of Macrophages Extracellular Traps,” Journal of Hazardous Materials 452 (2023): 131236, 10.1016/j.jhazmat.2023.131236. 36958159

